# Type 2 Diabetes Mellitus–Related Mortality in the United States, 1999 to 2023

**DOI:** 10.1016/j.jacadv.2025.101882

**Published:** 2025-06-18

**Authors:** Mushood Ahmed, Eeshal Zulfiqar, Aimen Shafiq, Maryam Shahzad, Tallal Mushtaq Hashmi, Raheel Ahmed, Jamal S. Rana, Stephen Sidney, Stephen J. Greene, Robert J. Mentz, Marat Fudim, Gregg C. Fonarow

**Affiliations:** a Rawalpindi Medical University, Rawalpindi, Pakistan; b Dow University of Health Sciences, Karachi, Pakistan; c Department of Cardiology, Royal Brompton Hospital, London, United Kingdom; d National Heart and Lung Institute, Imperial College London, London, United Kingdom; e Division of Cardiology, Kaiser Permanente Northern California, Oakland, California, USA; f Division of Research, Kaiser Permanente Northern California, Oakland, California, USA; g Medical University of South Carolina, Charleson, North Carolina, USA; h Division of Cardiology, Duke University Medical Center, Durham, North Carolina, USA; i Duke Clinical Research Institute, Durham, North Carolina, USA; j Ahmanson-UCLA Cardiomyopathy Center, Division of Cardiology, University of California-Los Angeles, Los Angeles, California, USA

**Keywords:** cardiovascular, CDC WONDER, type 2 diabetes mellitus, mortality

## Abstract

**Background:**

The prevalence of type 2 diabetes mellitus (T2DM) has increased in the United States, contributing significantly to morbidity and mortality.

**Objectives:**

This study analyzes trends in T2DM-related mortality focusing on demographic and regional disparities.

**Methods:**

The Centers for Disease Control and Prevention Wide-Ranging Online Data for Epidemiologic Research database was utilized to extract death certificate data for adults aged 25 and older from 1999 to 2023. Age-adjusted mortality rates (AAMRs) per 100,000 persons were calculated. Temporal trends were assessed by calculating the annual percent change using Joinpoint regression analysis.

**Results:**

From 1999 to 2023, a total of 2,031,626 deaths were attributed to T2DM in the United States. The AAMR more than doubled from 21.54 per 100,000 in 1999 to 53.95 per 100,000 in 2023 with a pronounced increase between 2018 and 2021 (AAMR: 62.7 in 2021, annual percent change: 16.06%; 95% CI: 11.84-19.66). Males had considerably higher AAMR than females (68.82 vs 42.48 in 2023). Among racial and ethnic groups, Hispanic or Latino populations had the highest AAMR in 2023 (69.69), followed by non-Hispanic Black or African American (65.45), non-Hispanic other populations (53.7), and non-Hispanic White group (49.98). The Western region of the United States showed the highest AAMR (78.29), and rural areas consistently had higher mortality rates compared to urban areas (69.88 vs 55.32 in 2020). From 1999 to 2023, cardiovascular disease accounted for 626,706 deaths among adults with T2DM.

**Conclusions:**

T2DM-related mortality has increased substantially over the time in the United States, with a peak observed between 2018 and 2021, emphasizing the need for targeted interventions.

Diabetes mellitus (DM) remains an important public health issue in the United States. Over 37 million adults (∼15% of the U.S. adult population) are currently diagnosed, and an additional 8 million live with undiagnosed disease.^1^ Of these 34 million people, approximately 32 million have type 2 DM (T2DM).^2^ Alarming data from the Framingham cohort report a near doubling in the incidence of newly diagnosed diabetes in men and women,^3^ highlighting the growing burden of this condition. Diabetes is a major risk factor for cardiovascular disease (CVD), chronic kidney disease, peripheral artery disease, and cerebrovascular disease.^4^ Hence, it is one of the most significant contributors to morbidity and mortality within the nation, having immense impacts on health care systems as well as patient quality of life.

T2DM is a challenge to health care providers despite therapeutic advances in dealing with the disease and public health measures directed at improving glycemic control and preventing complications. This is particularly concerning considering the dramatic rise in the incidence and prevalence of the disease in the last 20 years.^5^ Diabetes complications may be preventable, but several factors like health care inequities, lifestyle, and socioeconomic status, tend to have an adverse impact on many individuals.^2^ Considering the large scale and the significance of the problem, it is essential to understand the long-term diabetes-associated mortality trends for the assessment of current measures and planning future public health strategies.

This study uses data from the Centers for Disease Control and Prevention Wide-Ranging Online Data for Epidemiologic Research (CDC WONDER) database to analyze trends in mortality associated with T2DM in the United States from 1999 to 2023. The analysis stratifies findings by age, sex, race, and geographic location to determine the demographic disparities associated with T2DM-related mortality. Furthermore, a sensitivity analysis was conducted to identify T2DM-associated CVD mortality over the study period (1999-2023).

## Methods

### Study setting and population

Deaths in the United States related to T2DM were extracted from the CDC WONDER database.^6^ CDC WONDER is an exhaustive repository of death certificate data from the 50 states of the United States and the District of Columbia. The Multiple Cause-of-Death Public Use record death certificates were studied to identify records in which T2DM was mentioned as either a contributing or underlying cause of death on nationwide death certificates. Patients were identified using the International Classification of Diseases-10th Revision-Clinical Modification codes E11 for T2DM in individuals ≥25 years of age.^7^^,^^8^ A sensitivity analysis was conducted to determine T2DM-related CVD deaths where CVD (I00-I99) was listed as the underlying (primary) cause of death, and T2DM was listed as the contributing cause of death.^7^ Institutional Review Board approval was not required for this study, as we used a publicly available, deidentified data set provided by the government. The study adheres to the reporting standards outlined in the Strengthening the Reporting of Observational Studies in Epidemiology guidelines.^9^

### Data abstraction

Data on T2DM-related deaths and population sizes were extracted. Demographics (sex, race/ethnicity, and age) and regional information (urban-rural and state) were extracted from 1999 to 2023. Race/ethnicities were delineated as non-Hispanic (NH) White, NH Black or African American, NH others (NH Asian or Pacific Islander, NH American Indian or Alaska Native, etc), and Hispanics or Latinos.^10^ Trends in mortality from T2DM were evaluated based on state-specific variations, U.S. census regions (Northeast, Midwest, South, West), and county-level urbanization classifications. Counties were categorized as rural (micropolitan, noncore regions) or urban (large central metro, large fringe metro, medium metro, small metro) based on the 2013 National Center for Health Statistics Urban-Rural Classification Scheme.^11^

### Statistical analysis

Crude and age-adjusted mortality rates per 100,000 population were determined. Crude mortality rates (CMRs) were determined by dividing the number of T2DM-related deaths by the corresponding U.S. population of that year. Age-adjusted mortality rates (AAMRs) were calculated by standardizing the T2DM-related deaths to the 2000 U.S. population as previously described.^12^ The Joinpoint Regression Program (Joinpoint V 5.1.0.0, National Cancer Institute) was used to determine trends in AAMRs and CMRs using annual percent change (APC). This method identifies significant changes in AAMRs and CMRs over time by fitting log-linear regression models where temporal variation occurred. APCs with 95% CIs for the AAMRs and CMRs were calculated at the identified line segments linking join points using the Monte Carlo permutation test. APCs were considered increasing or decreasing if the slope describing the change in mortality was significantly different from zero using 2-tailed *t*-test. Statistical significance was set at *P* < 0.05.

## Results

### Overall

**Figure 1:**
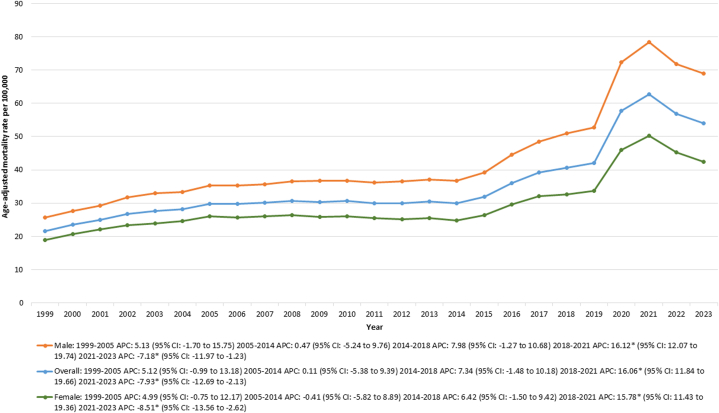
Type 2 Diabetes Mortality by Sex in the United States, 1999 to 2023 Age-adjusted mortality rates (AAMRs) per 100,000 individuals are shown for overall and sex-stratified populations in the United States from 1999 to 2023. APC = annual percent change.

From 1999 to 2023, there were 2,031,626 deaths in the United States attributed to T2DM. The AAMR increased from 21.54 in 1999 to 53.95 in 2023. Between 1999 and 2018, the AAMR had an overall increasing trend, followed by a pronounced increase from 40.57 to 62.72 between 2018 and 2021 (APC: 16.06% [95% CI: 11.84-19.66; *P* < 0.001]). This was followed by a decline in AAMR from 62.72 in 2021 to 53.95 in 2023 (APC: −8.51% [95% CI: −13.56 to −2.62; *P* = 0.03]) (Supplemental Table 1, Figure 1,Central Illustration).

**Central Illustration:**
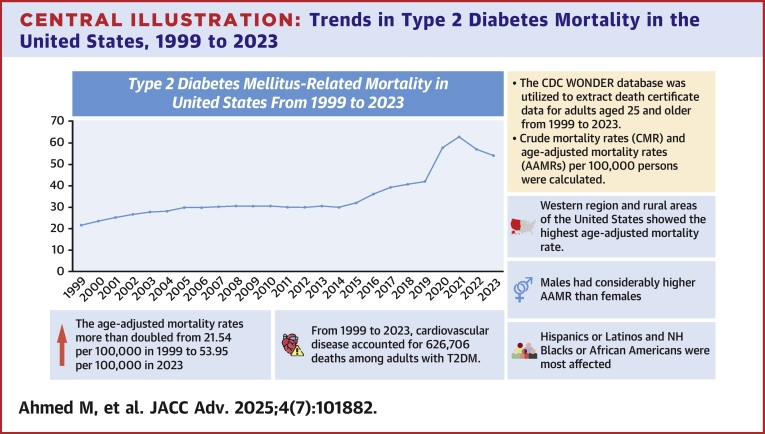
Trends in Type 2 Diabetes Mortality in the United States, 1999 to 2023 This central illustration summarizes the trends in age-adjusted mortality rates (AAMRs) related to type 2 diabetes mellitus in the United States from 1999 through 2023. The data highlight persistent disparities based on sex, race/ethnicity, and urbanization status. Despite advancements in diabetes care, mortality remains high, particularly among certain vulnerable populations. NH = non-Hispanic; T2DM = type 2 diabetes mellitus.

### T2DM-related AAMR stratified by sex

Throughout the study period, males consistently had a considerably higher AAMR than females. For males, the AAMR increased from 25.6 in 1999 to 68.88 in 2023, while for females, it rose from 18.83 in 1999 to 42.48 in 2023. Both groups showed a comparable AAMR trend between 1999 and 2018. From 2018 to 2021, the AAMR for males increased from 50.93 to 78.48 (APC: 16.12% [95% CI: 12.07-19.74; *P* < 0.001]), then declined to 68.88 in 2023 (APC: −7.18% [95% CI: −11.97 to −1.23; *P* = 0.04]). For females, the AAMR rose from 32.54 to 50.21 between 2018 and 2021 (APC: 15.78% [95% CI: 11.43-19.36; *P* < 0.001]), followed by a decrease to 42.48 in 2023 (APC: −8.51% [95% CI: −13.56 to −2.62; *P* = 0.02]) (Supplemental Tables 2 and 3).

### T2DM-related AAMR stratified race/ethnicity

Over the study period, the highest AAMR was observed in the Hispanic or Latino group, and NH Black or African American group.

For the Hispanic or Latino populations, the AAMR increased from 22.75 in 1999 to 51.9 in 2018 (APC: 3.66% [95% CI: 2.63-4.60; *P* = 0.0004]), followed by a further increase to 90.6 in 2021 (APC: 23.62% [95% CI: 16.09-28.77; *P* < 0.001]). However, the AAMR then decreased to 69.69 in 2023 (APC: −15.63% [95% CI: −21.66 to −8.41; *P* = 0.0004]).

For the Black or African American population, AAMR increased from 27.56 in 1999 to 39.5 in 2005 (APC: 5.78% [95% CI: 2.31-21.19; *P* = 0.003]), then remained stable till 2015. However, from 2015 to 2021, the AAMR rose from 39.05 to 73.87 (APC: 11.93% [95% CI: 9.53-20.00; *P* = 0.001]), followed by stability till 2023.

**Figure 2:**
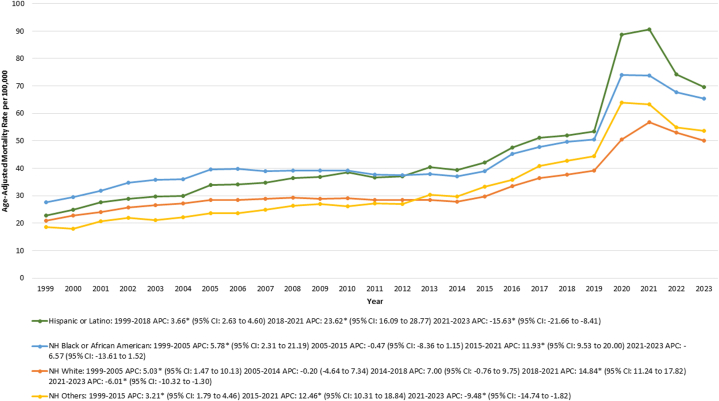
Type 2 Diabetes Mortality by Race/Ethnicity in the United States, 1999 to 2023 This figure presents age-adjusted mortality rates (AAMRs) per 100,000 individuals stratified by race/ethnicity, including non-Hispanic (NH) White, NH Black, Hispanic, and NH others, from 1999 to 2023. Abbreviation as in Figure 1.

For the NH other populations, the AAMR increased from 18.6 in 1999 to 33.23 in 2015 (APC: 3.21% [95% CI: 1.79-4.46; *P* = 0.002]). This upward trend continued as the AAMR rose to 63.22 in 2021 (APC: 12.46% [95% CI: 10.31-18.84; *P* = 0.0004]). However, from 2021 to 2023, the AAMR experienced a decline, decreasing to 53.7 (APC: −9.48% [95% CI: −14.74 to −1.82; *P* = 0.02]). Lastly, for the NH White population, the AAMR increased from 20.84 in 1999 to 28.54 in 2005 (APC: 5.03% [95% CI: 1.47-10.13; *P* = 0.04]). The AAMR then remained stable between 2005 and 2018. From 2018 to 2021, the AAMR saw an increase from 37.76 to 56.81 (APC: 14.84% [95% CI: 11.24-17.82; *P* < 0.001]). However, in the subsequent period from 2021 to 2023, the AAMR decreased to 49.98 (APC: −6.01% [95% CI: −10.32 to −1.30; *P* = 0.04]) (Supplemental Table 4, Figure 2).

### T2DM-related AAMR stratified by geographical region

#### Statewide

Throughout the study period, significant statewide variation in T2DM-related mortality was observed. From 1999 to 2020, states falling within the top 90th percentile for mortality rates included West Virginia, Oregon, and California, while those in the bottom 10th percentile were Nevada, Massachusetts, and Louisiana. In the subsequent period from 2021 to 2022, the states with the highest mortality rates were California, Wyoming, and Oklahoma, whereas Delaware, Connecticut, and Rhode Island ranked in the lowest 10th percentile. For the year 2023, the states in the top 90th percentile once again included Wyoming, California, and Oregon, while Delaware, Connecticut, and Rhode Island remained in the bottom 10th percentile (Supplemental Table 5).

#### Census region

From 1999 to 2023, the highest T2DM-related mortality rates were observed in the Western region, followed by the Midwest, the South, and lastly, the Northeastern region.

For the Western region, the AAMR increased from 19.07 in 1999 to 58.31 in 2018 (APC: 5.44% [95% CI: 4.83-6.01; *P* < 0.001]), followed by a further increase to 93.57 in 2021 (APC: 18.23% [95% CI: 13.59-20.96 *P* < 0.001]). The AAMR then declined to 78.29 in 2023 (APC: −8.78% [95% CI: −13.37 to −4.25; *P* = 0.0004]).

For the Midwest, the AAMR increased from 27.14 in 1999 to 37.04 in 2005 (APC: 4.75% [95% CI: 2.06-13.04; *P* < 0.001]), followed by stability until 2015. However, from 2015 to 2021, the AAMR rose from 35.41 to 65.59 (APC: 11.26% [95% CI: 9.36-17.10; *P* < 0.001]) and then remained stable till 2023.

For the Southern region, the AAMR increased from 21.03 in 1999 to 29.33 in 2005 (APC: 5.36% [95% CI: 2.49-9.70; *P* = 0.03]), followed by stability until 2018. The AAMR then rose from 35.71 in 2018 to 55.73 in 2021 (APC: 16.41% [95% CI: 12.41-19.50; *P* < 0.001]), followed by a decrease to 49.13 in 2023 (APC: −6.71% [95% CI: −10.85 to −2.43; *P* = 0.014]).

Lastly, for the Northeastern region, the AAMR remained stable between 1999 and 2017, then experienced an increase from 23.48 in 2017 to 37.42 in 2020 (APC: 19.80% [95% CI: 11.51-24.14; *P* = 0.006]). Following this period, the AAMR remained stable till 2023 (Supplemental Table 6).

#### Urban-rural

**Figure 3:**
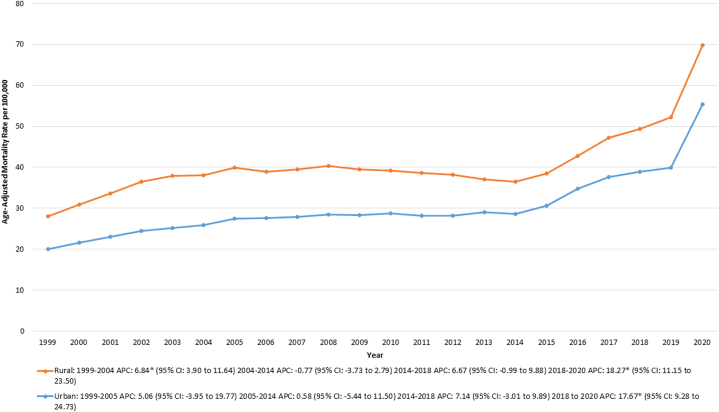
Type 2 Diabetes Mortality by Urbanization Level, 1999 to 2020 This figure presents age-adjusted mortality rates (AAMRs) per 100,000 individuals across different levels of urbanization (urban vs rural) in the United States from 1999 to 2020.∗Data for urbanization AAMRs were unavailable for 2021 to 2023. Abbreviation as in Figure 1.

Over the study period, rural areas averaged a considerably higher AAMR than urban areas. For rural areas, the AAMR increased from 28.8 in 1999 to 38.01 in 2004 (APC: 6.84% [95% CI: 3.90-11.64; *P* = 0.01]), and then remained stable until 2018. This was followed by a rise from 49.37 in 2018 to 69.88 in 2020 (APC: 18.27% [95% CI: 11.15-23.50; *P* < 0.001]). Similarly, for urban areas, the AAMR remained stable from 1999 to 2018, followed by an increase from 38.85 in 2018 to 55.32 in 2020 (APC: 17.67% [95% CI: 9.28-24.73; *P* < 0.001]) (Supplemental Table 7, Figure 3).

### T2DM-related CVD mortality

**Figure 4:**
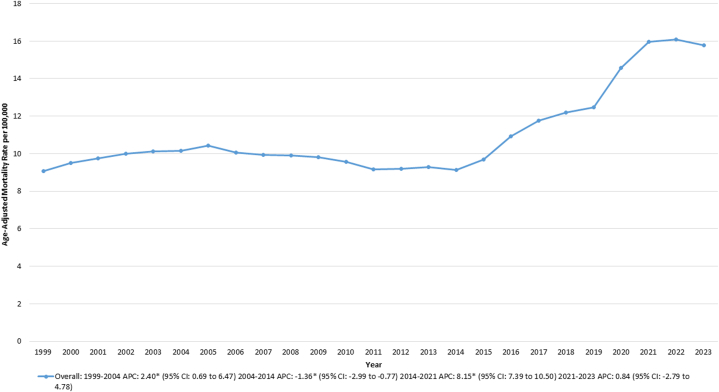
Cardiovascular Mortality Among Adults With Type 2 Diabetes, 1999 to 2023 This figure shows age-adjusted mortality rates (AAMRs) per 100,000 individuals for cardiovascular disease mortality among adults diagnosed with type 2 diabetes in the United States from 1999 to 2023. Abbreviation as in Figure 1.

From 1999 to 2023, a total of 626,706 T2DM-associated CVD deaths were recorded in the United States. The AAMR increased from 9.08 in 1999 to 10.14 in 2004 (APC: 2.4% [95% CI: 0.96-6.47; *P* = 0.006]), followed by a decrease to 9.13 in 2014 (APC: −1.36% [95% CI: −2.95 to −0.79; *P* = 0.006]). The AAMR then increased to 15.96 in 2021 (APC: 8.15% [95% CI: 7.40-10.61; *P* = 0.004]) followed by stability till 2023 (Supplemental Table 8, Figure 4).

## Discussion

This retrospective analysis of mortality data from the CDC WONDER database highlights important findings. The AAMR for T2DM in the United States increased over the 2 decades, reaching a peak during the COVID-19 pandemic and ultimately declining from 2021 to 2023. Males consistently exhibited higher AAMRs than females throughout the study period. Among racial/ethnic groups, the highest AAMRs were recorded in the Hispanic or Latino populations and NH Blacks or African Americans. Lastly, substantial geographic variations were identified, with Western region having the highest AAMR. Additionally, rural areas consistently exhibited higher T2DM-related AAMRs compared to urban areas.

The rise in mortality observed in our study can be attributed to several factors. Patients with T2DM continue to experience higher rates of all-cause and cardiovascular mortality, with CVD remaining the leading cause of death in this population.^13^^,^^14^ This occurs amid the obesity epidemic and the increasing prevalence of hypertension, hyperlipidemia, obesity, smoking, and physical inactivity.^15^, ^16^, ^17^, ^18^ Data from the National Health and Nutrition Examination Survey indicate that managing these modifiable risk factors remains suboptimal in diabetes patients, with only 56.2% achieving target low-density lipoprotein cholesterol (≤100 mg/dL [2.59 mmol/L]), 51.1% maintaining blood pressure at goal (≤130/80 mm Hg), and 52.5% meeting A1c targets (<7.0% [<53 mmol/mol]).^19^ The rise in T2DM mortality from 2018 to 2021 falls within the COVID-19 pandemic, which disproportionately affected patients with chronic conditions like T2DM. Patients with diabetes were more likely to experience the worst outcomes of COVID-19,^20^, ^21^, ^22^ including hospitalization and death, which partly contributed to the observed increase in mortality rates. Moreover, disruptions in health care access and management of chronic conditions during the pandemic likely contributed to poor glycemic control and exacerbated complications.^23^^,^^24^ The decline from 2021 onward potentially reflects improvement in pandemic management, increased rates of vaccination, and reinstatement of normal health care provision.^25^^,^^26^ However, the lingering effects of the pandemic on chronic disease management, particularly for T2DM patients, will require further study.

The persistent sex disparity noticed in this study, with males showing consistent and higher mortality rates than females, matches previous epidemiological studies.^27^ There was also racial and ethnic disparity concerning T2DM-related deaths. Grossly imbalanced AAMR rates among Hispanic and NH Black populations reflect the relatively higher burden of T2DM and its complications within these populations. This is consistent with national data showing greater rates of myocardial infarctions, heart failure, and stroke in the Black population and Hispanics which are often caused by underlying diabetes.^28^ Additionally, social determinants of health that may significantly influence these disparities include socioeconomic status, access to care, and health literacy. Further complicating the issue are factors such as delayed diagnosis, less aggressive treatment, and comorbidities that could be higher in certain populations which will also result in poorer outcomes.^29^, ^30^, ^31^ Nationally, NH Black and Hispanic patients are less likely to receive recommended diabetes preventive care, including hemoglobin A1c testing, cholesterol and blood pressure screening, and retinal examinations.^19^^,^^32^

In the past few years, many novel drugs such as sodium-glucose cotransporter-2 inhibitors^33^ and glucagon-like peptide-1 receptor agonists have established themselves as guideline-recommended therapies for improving cardiovascular outcomes in patients with T2DM.^34^ However, unequal access to these therapies limits these benefits in the case of racial and ethnic minorities.^35^, ^36^, ^37^ This may mean that access to more effective agents is contributing to continuous differences in mortality among NH Black populations and Hispanics.^35^^,^^38^ In addition, NH Black individuals with diabetes are at a higher risk of complications such as end-stage renal disease^39^^,^^40^ and amputations^41^^,^^42^ both of which significantly contribute to the increased death rate observed in our study. Additionally, barriers such as expensive medication, the absence of health insurance, and inequalities in access to care further confine the utilization of such treatments in minorities.^43^ Moreover, implicit provider biases may affect the providers' likelihood of ordering these modern therapies.^44^ This underutilization of efficacious therapies accelerates the already established discrepancies in deaths due to diabetes and draws more importance to more equitable mechanisms for health care service delivery.

The highest rate of T2DM-related deaths was seen in California, Wyoming, and Oregon. In contrast, Delaware, Connecticut, and Rhode Island registered the lowest T2DM death rates. The higher mortality rate in these Western regions could be due to a greater prevalence of diabetes and lesser accessibility to health services in the rural counties.^45^, ^46^, ^47^, ^48^ The observed regional disparities in T2DM-related mortality are likely attributable to a combination of social, structural, and health care system factors. Differences in access to high-quality care, availability of endocrinologists, affordability of newer antidiabetic medications, and health insurance coverage can significantly impact disease management and outcomes across states. Cultural dietary patterns, public health policies, and variable levels of investment in chronic disease prevention programs may further contribute to these disparities. Additionally, differences in the prevalence of comorbid conditions such as obesity, hypertension, and CVD across regions likely compound the risk. One of the major obstacles that exist for many minority patients in terms of limited access to preventive services and increased likelihood of complications is a lack of proper health insurance.^47^^,^^49^ Public health efforts in regions with high mortality should be targeted at enhancing preventive care, access to health care services, and lifestyle issues such as diet and exercise.

Our analysis also reveals that mortality from T2DM in the rural regions was higher compared to that in the urban regions, aligning with previous reports.^46^^,^^50^ In general, access to health services for the rural population is greatly impeded by the lack of health facilities and a smaller percentage of preventive care visits, with overall greater rates of poverty and unemployment.^51^^,^^52^ Additionally, most of the rural populations may not have direct access to endocrinologists, which could increase complications or delay proper diabetes management. Policies should be used to improve the rural infrastructure of health care, increase the number of health care workers, and expand telemedicine services so that patients with T2DM receive timely care.^47^^,^^52^

Finally, the large number of T2DM-related CVD deaths during the study period shows that diabetes and cardiovascular outcomes are closely related.^7^^,^^53^^,^^54^ Diabetes is a major risk factor for atherosclerosis, coronary artery disease, and heart failure, and T2DM patients often have coexisting conditions like hypertension and dyslipidemia, which further increases their risk of CVD events.^55^ The notable rise in CVD-related deaths among T2DM patients from 2018 to 2021 aligns with the pandemic period, where disruptions in care may have exacerbated cardiovascular risk. These findings support the need for integrated care models addressing both diabetes and cardiovascular risk through aggressive risk factor modification and regular cardiovascular screening of T2DM patients.

To bridge the mortality disparities caused by diabetes, public health agencies and policymakers should remove barriers that high-risk populations face in accessing diabetes care. Insurance coverage programs along with lower out-of-pocket costs for newer diabetes agents are a significant policy initiative that would prove essential for promoting equitable care.^56^^,^^57^ Culturally tailored public health interventions, such as community-based education programs that concentrate on diabetes self-management, nutrition, and physical activity, have been shown to improve outcomes in many populations.^58^, ^59^, ^60^ These interventions should become part of broader national strategies to reduce the burden of diabetes and its complications in underserved and at-risk communities.

### Study limitations

Some limitations need to be considered while interpreting the study findings. The study relied on International Classification of Diseases codes and death certificates which may have led to the misclassification of cause of death. Many individuals can have diabetes but if T2DM is not the primary cause of death, the treating physician might not mention it as a contributing cause of death on the death certificates. This can lead to an underestimation of the true burden of T2DM-related mortality. The errors in the completion of death certificates can also limit mortality analyses.^61^ The database lacks information on specific disease characteristics, including vital signs, lab results, echocardiographic findings, and genetic testing. Additionally, it does not include information on social determinants of health, which could affect access to health care and potentially influence racial and ethnic disparities in mortality related to T2DM. It is important to mention that the CDC WONDER database does not include mortality data for U.S. citizens in Puerto Rico, Guam, and American Samoa; therefore, these regions were not included in our study. Furthermore, although JoinPoint regression identified a significant increase in mortality from 2018 to 2021, likely reflecting the impact of the COVID-19 pandemic, we were unable to quantify the exact contribution of the pandemic to this trend. The CDC WONDER database does not provide detailed data on pandemic-related factors that may have influenced mortality rates during this period. Finally, although we presented comparative trends across sex, race, ethnicity, and geographic groups, our analysis descriptively compared AAMRs and CMRs obtained from the CDC WONDER database and did not include formal statistical testing between subgroups. This limits the strength of conclusions regarding the statistical significance of observed differences and may not fully account for potential confounders. However, this descriptive approach has been utilized in previously published studies using the CDC WONDER database and aligns with established epidemiologic reporting practices.^62^

## Conclusions

T2DM-related mortality increased over time in the United States reaching a peak in 2021. Males consistently had higher AAMRs compared to females. The Hispanic or Latino and NH Black or African American populations, Western region, and rural areas showed the highest mortality rates. Additionally, CVD was a major contributor to T2DM-related deaths, with a pronounced rise in CVD mortality observed after 2014. These findings emphasize the need for targeted interventions to address the rising burden of diabetes-related mortality and its disparities across different demographic and geographic groups.

Perspectives **COMPETENCY IN MEDICAL KNOWLEDGE:** Our study demonstrated a more than 2-fold rise in T2DM-related mortality in the United States from 1999 to 2023. A pronounced increase was observed during the COVID-19 pandemic. Mortality rates are higher among men, minorities, and rural areas.

**TRANSLATIONAL OUTLOOK:** Targeted interventions are required to reduce T2DM mortality and improve health care access in vulnerable communities.

## Funding support and author disclosures

Dr Fonarow has received personal fees from Abbott, Amgen, AstraZeneca, Bayer, Boehringer Ingelheim, Cytokinetics, Eli Lilly, Johnson & Johnson, Medtronic, Merck, Novartis, and Pfizer outside the submitted work. Dr Fudim has received personal fees from Alleviant, Ajax, Alio Health, Alleviant, Artha, Audicor, Axon Therapies, Bayer, Bodyguide, Bodyport, Boston Scientific, Broadview, Cadence, Cardioflow, Cardionomics, Coridea, CVRx, Daxor, Deerfield Catalyst, Edwards Lifesciences, Echosens, EKO, Feldschuh Foundation, Fire1, FutureCardia, Galvani, Gradient, Hatteras, HemodynamiQ, Impulse Dynamics, Intershunt, Medtronic, Merck, NIMedical, NovoNordisk, NucleusRx, NXT Biomedical, Orchestra, Pharmacosmos, PreHealth, Presidio, Procyreon, ReCor, Rockley, SCPharma, Shifamed, Splendo, Summacor, SyMap, Verily, Vironix, Viscardia, and Zoll; and has received grants from the 10.13039/100000002National Institutes of Health, Doris Duke, outside the submitted work. Dr Mentz has received research support and honoraria from Abbott, American Regent, Amgen, AstraZeneca, Bayer, Boehringer Ingelheim, Boston Scientific, Cytokinetics, Fast BioMedical, Gilead, Innolife, Eli Lilly, Medtronic, Medable, Merck, Novartis, Novo Nordisk, Pfizer, Pharmacosmos, Relypsa, Respicardia, Roche, Rocket Pharmaceuticals, Sanofi, Verily, Vifor, Windtree Therapeutics, and Zoll. Dr Greene has received research support from the Duke University Department of Medicine Chair's Research Award, American Heart Association, Amgen, AstraZeneca, Boehringer Ingelheim, Bristol Myers Squibb, Cytokinetics, Merck, Novartis, Otsuka, Pfizer, and Sanofi; has served on advisory boards or as consultant for Amgen, AstraZeneca, Bayer, Boehringer Ingelheim, Bristol Myers Squibb, Corcept, Corteria Pharmaceuticals, CSL Vifor, Cytokinetics, Eli Lilly, Lexicon, Merck, Novo Nordisk, Otsuka, Roche Diagnostics, Sanofi, scPharmaceuticals, Tricog Health, and Urovant Pharmaceuticals; and has received speaker fees from AstraZeneca, Bayer, Boehringer Ingelheim, Cytokinetics, Lexicon, and Roche Diagnostics. All other authors have reported that they have no relationships relevant to the contents of this paper to disclose.
