# Supplementary material for: Type 2 Diabetes Mellitus–Related Mortality in the United States, 1999 to 2023
Source: JACC Adv. 2025 Jun 18;4(7):101882. doi: 10.1016/j.jacadv.2025.101882 (PMC12212274; doi:10.1016/j.jacadv.2025.101882)
Supplement: Supplementary data [file mmc1.pdf]

Supplementary Appendix

Supplemental Table 1: Type 2 Diabetes Mellitus (T2DM) related deaths, Stratified by Sex and Race in the United States, 1999 to 2023

	Deaths						
**Year**	Overall	Female	Male	NH Black or African American	White	Hispanic or Latino	NH Others
**1999**	37991	20290	17701	4031	31136	1879	864
**2000**	41853	22505	19348	4392	34336	2147	880
**2001**	45383	24409	20974	4801	36831	2543	1081
**2002**	49162	26026	23136	5346	39639	2817	1228
**2003**	51633	26977	24656	5639	41542	3082	1262
**2004**	53417	27970	25447	5748	42897	3275	1384
**2005**	57678	30081	27597	6486	45624	3908	1569
**2006**	58663	30200	28463	6684	46058	4135	1679
**2007**	60591	31256	29335	6715	47530	4439	1833
**2008**	63130	32145	30985	6965	49107	4910	2038
**2009**	63807	31921	31886	7187	49018	5276	2226
**2010**	65319	32781	32538	7343	49943	5717	2223
**2011**	65791	32723	33068	7322	50081	5821	2482
**2012**	67399	33019	34380	7550	50875	6213	2617
**2013**	70206	34096	36110	7922	51909	7129	3139
**2014**	70584	33796	36788	8014	51703	7426	3280
**2015**	77339	36905	40434	8735	55946	8498	3920

**2016**	89210	41949	47261	10452	64040	10044	4449
**2017**	99374	46529	52845	11441	70907	11370	5410
**2018**	105303	48229	57074	12253	74810	12117	5927
**2019**	111411	50850	60561	12789	78977	12969	6461
**2020**	155630	70405	85225	19475	103078	22933	9856
**2021**	165420	73946	91474	19093	110897	24183	10953
**2022**	156826	70657	86169	17937	108256	20125	10131
**2023**	148506	66284	82222	17224	102147	18875	9880

NH, non-Hispanic

Supplemental Table 2: Annual percent change (APC) of T2DM-related age-adjusted mortality rates per 100,000 in the United States, 1999 to 2023

Year Interval	APC (95% confidence interval)
**Overall**	
1999-2005	5.12 (-0.99 to 13.18)
2005-2014	0.11 (-5.38 to 9.39)
2014-2018	7.34 (-1.48 to 10.18)
2018-2021	16.06* (11.84 to 19.66)
2021-2023	8.51* (-13.56 to -2.62)
**Male**	
1999-2005	5.13 (-1.70 to 15.75)
2005-2014	0.47 (-5.24 to 9.76)
2014-2018	7.98 (-1.27 to 10.68)
2018-2021	16.12* (12.07 to 19.74)
2021-2023	-7.18* (-11.97 to -1.23)
**Female**	
1999-2005	4.99 (-0.75 to 12.17)
2005-2014	-0.41 (-5.82 to 8.89)
2014-2018	7.98 (-1.27 to 10.68)
2018-2021	15.78* (11.43 to 19.36)
2021-2023	8.51* (-13.56 to -2.62)
**Black or African American**	

1999-2005	5.78* (2.31 to 21.19)
2005-2015	-0.47 (-8.36 to 1.15)
2015-2021	11.93* (9.53 to 20.00)
2021-2023	-6.57 (-13.61 to 1.52)
**White**	
1999-2005	5.03* (1.47 to 10.13)
2005-2014	-0.20 (-4.64 to 7.34)
2014-2018	7.00 (-0.76 to 9.75)
2018-2021	14.84* (11.24 to 17.82)
2021-2023	-6.01* (-10.32 to -1.30)
**Hispanic or Latino**	
1999-2018	3.66* (2.63 to 4.60)
2018-2021	23.62* (16.09 to 28.77)
2021-2023	-15.63* (-21.66 to -8.41)
**NH Others**	
1999-2015	3.21* (1.79 to 4.46)
2015-2021	12.46* (10.31 to 18.84)
2021-2023	-9.48* (-14.74 to -1.82)
**Northeast**	
1999-2017	0.068 (-0.81 to 0.76)
2017-2020	19.80* (11.51 to 24.14)
2020-2023	-3.65 (-10.97 to 0.64)
**Midwest**	

1999-2005	4.75* (2.06 to 13.04)
2005-2015	-1.00 (-6.86 to 0.19)
2015-2021	11.26* (9.36 to 17.10)
2021-2023	-6.37 (-12.90 to 0.93)
**South**	
1999-2005	5.36* (2.49 to 9.70)
2005-2014	-1.29 (-5.50 to 4.33)
2014-2018	7.38 (-1.27 to 10.61)
2018-2021	16.41* (12.41 to 19.50)
2021-2023	-6.71* (-10.85 to -2.43)
**West**	
1999-2018	5.44* (4.83 to 6.01)
2018-2021	18.23* (13.59 to 20.96)
2021-2023	-8.78* (-13.37 to -4.25)
**Urban**	
1999-2005	5.06 (-3.95 to 19.77)
2005-2014	0.58 (-5.44 to 11.50)
2014-2018	7.14 (-3.01 to 9.89)
2018-2020	17.67* (9.28 to 24.73)
**Rural**	
1999-2004	6.84* (3.90 to 11.64)
2004-2014	-0.77 (-3.73 to 2.79)
2014-2018	6.67 (-0.99 to 9.88)

2018-2020	18.27* (11.15 to 23.50)

* Significant values

Supplemental Table 3: Overall and Sex-Stratified T2DM-Related Age-Adjusted Mortality Rates per 100,000 in the United States, 1999 to 2023

	Age-Adjusted Rate (95% CI)		
**Year**	**Male**	**Female**	**Overall**
**1999**	25.6 (25.22 - 25.99)	18.83 (18.57 - 19.09)	21.54 (21.32 - 21.76)
**2000**	27.56 (27.17 - 27.96)	20.66 (20.39 - 20.93)	23.45 (23.23 - 23.68)
**2001**	29.29 (28.89 - 29.7)	22.13 (21.85 - 22.41)	25.01 (24.78 - 25.24)
**2002**	31.71 (31.29 - 32.12)	23.35 (23.07 - 23.64)	26.66 (26.43 - 26.9)
**2003**	32.89 (32.47 - 33.31)	23.89 (23.6 - 24.18)	27.56 (27.32 - 27.8)
**2004**	33.32 (32.91 - 33.74)	24.52 (24.23 - 24.81)	28.1 (27.86 - 28.34)
**2005**	35.2 (34.77 - 35.62)	26 (25.71 - 26.3)	29.77 (29.53 - 30.01)
**2006**	35.33 (34.91 - 35.75)	25.65 (25.36 - 25.94)	29.68 (29.44 - 29.92)
**2007**	35.59 (35.17 - 36)	26.08 (25.79 - 26.38)	30.07 (29.83 - 30.31)
**2008**	36.6 (36.18 - 37.01)	26.36 (26.07 - 26.65)	30.65 (30.41 - 30.89)
**2009**	36.64 (36.23 - 37.05)	25.83 (25.55 - 26.12)	30.35 (30.12 - 30.59)
**2010**	36.78 (36.37 - 37.19)	26.1 (25.81 - 26.38)	30.58 (30.34 - 30.82)
**2011**	36.15 (35.75 - 36.55)	25.47 (25.19 - 25.75)	29.99 (29.76 - 30.23)
**2012**	36.49 (36.1 - 36.89)	25.04 (24.76 - 25.31)	29.92 (29.69 - 30.14)
**2013**	37.08 (36.69 - 37.47)	25.44 (25.16 - 25.71)	30.42 (30.19 - 30.65)
**2014**	36.68 (36.3 - 37.07)	24.71 (24.44 - 24.98)	29.88 (29.65 - 30.1)
**2015**	39.23 (38.84 - 39.61)	26.41 (26.14 - 26.69)	31.95 (31.72 - 32.18)
**2016**	44.62 (44.21 - 45.03)	29.53 (29.24 - 29.82)	36.08 (35.84 - 36.32)

**2017**	48.53 (48.11 - 48.95)	32.06 (31.76 - 32.36)	39.22 (38.98 - 39.47)
**2018**	50.93 (50.5 - 51.35)	32.54 (32.24 - 32.83)	40.57 (40.32 - 40.82)
**2019**	52.73 (52.3 - 53.15)	33.64 (33.34 - 33.94)	42.01 (41.76 - 42.26)
**2020**	72.44 (71.95 - 72.94)	46.01 (45.66 - 46.36)	57.7 (57.41 - 57.99)
**2021**	78.48 (77.96 - 79)	50.21 (49.84 - 50.57)	62.72 (62.42 - 63.03)
**2022**	71.83 (71.34 - 72.32)	45.3 (44.96 - 45.64)	56.9 (56.61 - 57.18)
**2023**	68.88 (68.4 - 69.36)	42.48 (42.15 - 42.81)	53.95 (53.68 - 54.23)

Supplemental Table 4: T2DM- Related Age-Adjusted Mortality Rates per 100,000, Stratified by Race in the United States, 1999 to 2023

	Age-Adjusted Rate (95% CI)			
**Year**	**NH Black or African American**	**NH White**	**Hispanic or Latino**	**NH Others**
**1999**	27.56 (26.7 - 28.42)	20.84 (20.6 - 21.07)	22.75 (21.68 - 23.81)	18.6 (17.3 - 19.89)
**2000**	29.6 (28.72 - 30.48)	22.78 (22.54 - 23.03)	24.84 (23.75 - 25.93)	17.88 (16.65 - 19.11)
**2001**	31.86 (30.95 - 32.77)	24.15 (23.91 - 24.4)	27.54 (26.43 - 28.65)	20.77 (19.48 - 22.06)
**2002**	34.75 (33.8 - 35.69)	25.73 (25.48 - 25.98)	28.82 (27.71 - 29.92)	21.97 (20.69 - 23.25)
**2003**	35.91 (34.96 - 36.86)	26.61 (26.36 - 26.87)	29.82 (28.73 - 30.92)	21.07 (19.86 - 22.27)
**2004**	36.09 (35.14 - 37.04)	27.21 (26.95 - 27.46)	29.97 (28.9 - 31.04)	22.18 (20.97 - 23.4)
**2005**	39.5 (38.52 - 40.48)	28.54 (28.28 - 28.81)	33.96 (32.85 - 35.06)	23.66 (22.45 - 24.88)
**2006**	39.81 (38.84 - 40.79)	28.42 (28.16 - 28.68)	34.12 (33.03 - 35.2)	23.61 (22.44 - 24.78)

**2007**	38.93 (37.98 - 39.88)	28.87 (28.61 - 29.13)	34.76 (33.7 - 35.82)	24.93 (23.75 - 26.11)
**2008**	39.06 (38.12 - 40)	29.35 (29.09 - 29.61)	36.37 (35.31 - 37.43)	26.43 (25.25 - 27.62)
**2009**	39.16 (38.23 - 40.08)	28.87 (28.61 - 29.13)	36.95 (35.91 - 37.98)	27.09 (25.93 - 28.26)
**2010**	39.12 (38.2 - 40.04)	29.03 (28.77 - 29.28)	38.61 (37.57 - 39.65)	26.12 (25 - 27.24)
**2011**	37.62 (36.73 - 38.5)	28.54 (28.29 - 28.8)	36.55 (35.57 - 37.52)	27.11 (26.01 - 28.2)
**2012**	37.45 (36.59 - 38.32)	28.43 (28.18 - 28.68)	37.14 (36.19 - 38.1)	26.98 (25.92 - 28.04)
**2013**	37.99 (37.13 - 38.85)	28.48 (28.23 - 28.73)	40.32 (39.35 - 41.28)	30.37 (29.28 - 31.45)
**2014**	37.17 (36.34 - 38.01)	27.93 (27.69 - 28.18)	39.36 (38.44 - 40.29)	29.62 (28.58 - 30.65)
**2015**	39.05 (38.21 - 39.89)	29.71 (29.46 - 29.96)	42.07 (41.15 - 43)	33.23 (32.17 - 34.29)
**2016**	45.24 (44.35 - 46.13)	33.48 (33.22 - 33.75)	47.61 (46.65 - 48.57)	35.79 (34.72 - 36.86)
**2017**	47.75 (46.85 - 48.65)	36.46 (36.18 - 36.73)	51.23 (50.26 - 52.2)	40.87 (39.76 - 41.98)

**2018**	49.56 (48.66 - 50.46)	37.76 (37.49 - 38.04)	51.9 (50.95 - 52.85)	42.71 (41.61 - 43.81)
**2019**	50.41 (49.51 - 51.3)	39.13 (38.85 - 39.41)	53.41 (52.46 - 54.35)	44.35 (43.25 - 45.44)
**2020**	74.1 (73.04 - 75.17)	50.58 (50.26 - 50.89)	88.66 (87.47 - 89.84)	64.03 (62.75 - 65.31)
**2021**	73.87 (72.79 - 74.95)	56.81 (56.47 - 57.16)	90.6 (89.42 - 91.79)	63.22 (62.02 - 64.42)
**2022**	67.8 (66.78 - 68.82)	52.96 (52.64 - 53.28)	74.14 (73.08 - 75.19)	54.8 (53.72 - 55.88)
**2023**	65.45 (64.45 - 66.45)	49.98 (49.67 - 50.29)	69.69 (68.67 - 70.72)	53.7 (52.63 - 54.77)

NH = non-Hispanic.

Supplemental Table 5: T2DM-Related Age-Adjusted Mortality Rates per 100,000, Stratified by States in the United States, 1999 to 2023

State	Age-Adjusted Rate (95% CI)		
	**1999-2020**	**2021-2022**	**2023**
**Alabama**	27.91 (27.53 - 28.29)	52.45 (50.88 - 54.03)	42.39 (40.42 - 44.37)
**Alaska**	32.88 (31.38 - 34.37)	51.92 (46.97 - 56.88)	38.84 (32.79 - 44.9)
**Arizona**	24.73 (24.42 - 25.04)	48.09 (46.85 - 49.32)	45.06 (43.4 - 46.72)
**Arkansas**	24.77 (24.32 - 25.22)	49.83 (47.85 - 51.81)	45.81 (43.15 - 48.47)
**California**	48.52 (48.33 - 48.72)	112.17 (111.31 - 113.03)	96.54 (95.42 - 97.66)
**Colorado**	33.86 (33.41 - 34.31)	70.96 (69.11 - 72.82)	58.91 (56.56 - 61.26)
**Connecticut**	18.15 (17.81 - 18.49)	20.97 (19.84 - 22.1)	18.48 (16.98 - 19.97)
**Delaware**	27.65 (26.79 - 28.5)	15.18 (13.38 - 16.99)	10.37 (8.33 - 12.41)
**District of Columbia**	19.26 (18.32 - 20.2)	33.88 (29.98 - 37.79)	31.6 (26.28 - 36.93)

**Florida**	20.85 (20.7 - 21)	40.38 (39.77 - 40.98)	36.94 (36.14 - 37.74)
**Georgia**	18.75 (18.5 - 18.99)	22.99 (22.22 - 23.77)	20.86 (19.83 - 21.9)
**Hawaii**	24.52 (23.88 - 25.16)	42.89 (40.38 - 45.41)	40.93 (37.51 - 44.35)
**Idaho**	42.39 (41.53 - 43.25)	92.14 (88.58 - 95.7)	89.65 (84.76 - 94.54)
**Illinois**	25.35 (25.12 - 25.57)	35.52 (34.7 - 36.35)	33.27 (32.15 - 34.38)
**Indiana**	40.68 (40.28 - 41.08)	65.21 (63.66 - 66.76)	50.8 (48.88 - 52.71)
**Iowa**	48.36 (47.77 - 48.94)	78.2 (75.82 - 80.57)	75.28 (72.02 - 78.55)
**Kansas**	35.74 (35.19 - 36.29)	60.08 (57.83 - 62.32)	52.8 (49.86 - 55.75)
**Kentucky**	44.29 (43.78 - 44.8)	94.35 (92.07 - 96.62)	80.07 (77.14 - 83.01)
**Louisiana**	15.82 (15.51 - 16.12)	26.94 (25.72 - 28.16)	22.54 (20.98 - 24.11)
**Maine**	34.12 (33.38 - 34.85)	53.43 (50.66 - 56.19)	50.05 (46.33 - 53.76)
**Maryland**	26.59 (26.24 - 26.94)	57.34 (55.83 - 58.85)	54.86 (52.8 - 56.92)

**Massachusetts**	14.62 (14.4 - 14.85)	27.91 (26.95 - 28.87)	26.64 (25.33 - 27.96)
**Michigan**	31.06 (30.78 - 31.33)	59.33 (58.16 - 60.5)	50.64 (49.13 - 52.15)
**Minnesota**	46.41 (45.94 - 46.87)	99.02 (96.97 - 101.06)	92.37 (89.62 - 95.13)
**Mississippi**	23.58 (23.13 - 24.04)	50.71 (48.63 - 52.79)	48.97 (46.1 - 51.85)
**Missouri**	30.17 (29.82 - 30.51)	46.22 (44.89 - 47.54)	43.26 (41.47 - 45.06)
**Montana**	36.41 (35.5 - 37.32)	66.75 (63.06 - 70.44)	71.06 (65.71 - 76.4)
**Nebraska**	46.63 (45.86 - 47.41)	77.12 (73.98 - 80.26)	73.11 (68.88 - 77.34)
**Nevada**	14.22 (13.82 - 14.62)	37.22 (35.45 - 38.99)	35.94 (33.53 - 38.35)
**New Hampshire**	30.88 (30.13 - 31.63)	44.43 (41.8 - 47.06)	39.3 (35.86 - 42.73)
**New Jersey**	17.59 (17.37 - 17.81)	23.48 (22.71 - 24.25)	20.59 (19.57 - 21.6)
**New Mexico**	32.83 (32.19 - 33.48)	64.85 (62.15 - 67.55)	58.58 (54.98 - 62.17)
**New York**	17.61 (17.47 - 17.76)	29.84 (29.26 - 30.42)	26.51 (25.74 - 27.28)

**North Carolina**	34.06 (33.75 - 34.36)	61.45 (60.26 - 62.65)	63.92 (62.22 - 65.62)
**North Dakota**	41.73 (40.57 - 42.89)	62.63 (58.15 - 67.11)	59.01 (52.83 - 65.2)
**Ohio**	47.95 (47.64 - 48.27)	63.74 (62.61 - 64.86)	55.98 (54.5 - 57.46)
**Oklahoma**	47.04 (46.48 - 47.61)	103.1 (100.53 - 105.68)	90.58 (87.2 - 93.97)
**Oregon**	48.98 (48.44 - 49.53)	95.34 (93.06 - 97.62)	96.4 (93.21 - 99.59)
**Pennsylvania**	32.6 (32.37 - 32.83)	52.96 (52.03 - 53.9)	49 (47.74 - 50.26)
**Rhode Island**	23.55 (22.86 - 24.24)	21.86 (19.77 - 23.96)	8.96 (7.18 - 11.03)
**South Carolina**	31.28 (30.86 - 31.69)	23.74 (22.7 - 24.78)	20.91 (19.57 - 22.26)
**South Dakota**	42.92 (41.84 - 44)	93.25 (88.2 - 98.29)	86.24 (79.42 - 93.05)
**Tennessee**	45.22 (44.79 - 45.64)	81.57 (79.86 - 83.27)	79.49 (77.13 - 81.84)
**Texas**	38.95 (38.73 - 39.17)	65.66 (64.83 - 66.49)	60.63 (59.51 - 61.74)
**Utah**	28.11 (27.5 - 28.73)	53.94 (51.53 - 56.34)	47.54 (44.39 - 50.68)

**Vermont**	40.17 (38.97 - 41.38)	67.06 (62.42 - 71.71)	62.74 (56.48 - 69)
**Virginia**	26.56 (26.26 - 26.86)	50.77 (49.56 - 51.98)	50.03 (48.35 - 51.7)
**Washington**	42.74 (42.33 - 43.15)	75.69 (74.1 - 77.27)	68.43 (66.32 - 70.53)
**West Virginia**	56.18 (55.38 - 56.99)	89.58 (86.31 - 92.85)	64.43 (60.53 - 68.33)
**Wisconsin**	38.32 (37.92 - 38.72)	85.61 (83.77 - 87.45)	80.01 (77.53 - 82.49)
**Wyoming**	44.25 (42.79 - 45.7)	110.92 (104.03 - 117.81)	99.8 (90.62 - 108.97)

Supplemental Table 6: T2DM-Related Age-Adjusted Mortality Rates per 100,000, Stratified by Census Region in the United States, 1999 to 2023

Census Region	Year	Age-Adjusted Rate (95% CI)
**Northeast**		
Northeast	1999	18.39 (17.96 - 18.83)
Northeast	2000	19.95 (19.5 - 20.4)
Northeast	2001	20.43 (19.98 - 20.89)
Northeast	2002	21.51 (21.05 - 21.98)
Northeast	2003	21.97 (21.51 - 22.44)
Northeast	2004	22.38 (21.92 - 22.85)
Northeast	2005	22.19 (21.73 - 22.65)
Northeast	2006	21.29 (20.84 - 21.75)
Northeast	2007	20.92 (20.47 - 21.36)
Northeast	2008	20.9 (20.46 - 21.34)
Northeast	2009	20.64 (20.2 - 21.08)
Northeast	2010	20.46 (20.02 - 20.89)
Northeast	2011	19.7 (19.27 - 20.12)
Northeast	2012	20.06 (19.63 - 20.48)
Northeast	2013	19.44 (19.03 - 19.85)
Northeast	2014	19.25 (18.84 - 19.66)
Northeast	2015	20.29 (19.87 - 20.71)
Northeast	2016	22.33 (21.89 - 22.76)

Northeast	2017	23.48 (23.04 - 23.92)
Northeast	2018	24.76 (24.31 - 25.21)
Northeast	2019	25.92 (25.47 - 26.38)
Northeast	2020	37.42 (36.87 - 37.96)
Northeast	2021	36.25 (35.71 - 36.79)
Northeast	2022	33.49 (32.98 - 33.99)
Northeast	2023	31.53 (31.04 - 32.02)
**Midwest**		
Midwest	1999	27.14 (26.65 - 27.64)
Midwest	2000	29.7 (29.18 - 30.21)
Midwest	2001	31.52 (30.99 - 32.05)
Midwest	2002	33.66 (33.12 - 34.21)
Midwest	2003	34.81 (34.26 - 35.36)
Midwest	2004	34.81 (34.26 - 35.35)
Midwest	2005	37.04 (36.48 - 37.6)
Midwest	2006	36.41 (35.85 - 36.96)
Midwest	2007	36.86 (36.31 - 37.42)
Midwest	2008	37.45 (36.89 - 38)
Midwest	2009	35.79 (35.25 - 36.33)
Midwest	2010	35 (34.47 - 35.53)
Midwest	2011	35.07 (34.54 - 35.59)
Midwest	2012	34.39 (33.87 - 34.9)
Midwest	2013	33.44 (32.93 - 33.94)

Midwest	2014	33.2 (32.7 - 33.7)
Midwest	2015	35.41 (34.9 - 35.92)
Midwest	2016	39.66 (39.12 - 40.2)
Midwest	2017	43.32 (42.77 - 43.88)
Midwest	2018	44.28 (43.72 - 44.84)
Midwest	2019	45.01 (44.46 - 45.57)
Midwest	2020	61.31 (60.66 - 61.95)
Midwest	2021	65.59 (64.91 - 66.27)
Midwest	2022	59.79 (59.15 - 60.42)
Midwest	2023	56.29 (55.67 - 56.91)
**South**		
South	1999	21.03 (20.67 - 21.39)
South	2000	23.04 (22.66 - 23.41)
South	2001	24.88 (24.49 - 25.27)
South	2002	26.48 (26.09 - 26.88)
South	2003	27.26 (26.86 - 27.66)
South	2004	27.33 (26.93 - 27.72)
South	2005	29.33 (28.93 - 29.74)
South	2006	29.29 (28.89 - 29.69)
South	2007	29.05 (28.66 - 29.45)
South	2008	28.79 (28.4 - 29.17)
South	2009	29.19 (28.8 - 29.58)
South	2010	29.02 (28.64 - 29.4)

South	2011	27.36 (27 - 27.73)
South	2012	27.18 (26.82 - 27.54)
South	2013	27.1 (26.74 - 27.45)
South	2014	26.12 (25.77 - 26.46)
South	2015	27.28 (26.94 - 27.63)
South	2016	31.57 (31.2 - 31.94)
South	2017	34.14 (33.76 - 34.52)
South	2018	35.71 (35.33 - 36.09)
South	2019	37.51 (37.13 - 37.9)
South	2020	50.6 (50.16 - 51.04)
South	2021	55.73 (55.26 - 56.2)
South	2022	50.96 (50.52 - 51.39)
South	2023	49.13 (48.7 - 49.56)
**West**		
West	1999	19.07 (18.61 - 19.53)
West	2000	20.27 (19.8 - 20.74)
West	2001	22.36 (21.88 - 22.85)
West	2002	24.12 (23.62 - 24.62)
West	2003	25.3 (24.8 - 25.81)
West	2004	27.36 (26.84 - 27.88)
West	2005	29.63 (29.1 - 30.16)
West	2006	30.92 (30.38 - 31.46)
West	2007	32.98 (32.43 - 33.53)

West	2008	35.57 (35.01 - 36.14)
West	2009	35.52 (34.96 - 36.08)
West	2010	37.81 (37.24 - 38.38)
West	2011	38.42 (37.86 - 38.99)
West	2012	38.75 (38.19 - 39.31)
West	2013	42.58 (42 - 43.16)
West	2014	41.89 (41.33 - 42.46)
West	2015	46.23 (45.65 - 46.82)
West	2016	51.51 (50.9 - 52.12)
West	2017	57.02 (56.38 - 57.65)
West	2018	58.31 (57.68 - 58.94)
West	2019	59.88 (59.25 - 60.51)
West	2020	82.35 (81.62 - 83.08)
West	2021	93.57 (92.78 - 94.37)
West	2022	83.37 (82.65 - 84.1)
West	2023	78.29 (77.58 - 78.99)

Supplemental Table 7: T2DM-Related Age-Adjusted Mortality Rates per 100,000 in United States stratified by Urban-Rural Classification, 1999-2020

	**Age-Adjusted Rate (95% CI)**	
**Year**	**Urban**	**Rural**
**1999**	19.97 (19.74 - 20.2)	28.1 (27.53 - 28.67)
**2000**	21.65 (21.41 - 21.89)	30.93 (30.34 - 31.52)
**2001**	23.01 (22.77 - 23.26)	33.55 (32.93 - 34.16)
**2002**	24.44 (24.19 - 24.69)	36.45 (35.81 - 37.09)
**2003**	25.17 (24.92 - 25.42)	37.97 (37.32 - 38.61)
**2004**	25.83 (25.58 - 26.08)	38.01 (37.37 - 38.66)
**2005**	27.46 (27.2 - 27.72)	39.98 (39.32 - 40.64)
**2006**	27.66 (27.4 - 27.92)	38.91 (38.26 - 39.55)
**2007**	27.95 (27.69 - 28.2)	39.54 (38.89 - 40.18)
**2008**	28.51 (28.26 - 28.77)	40.33 (39.68 - 40.98)
**2009**	28.38 (28.13 - 28.64)	39.55 (38.91 - 40.19)
**2010**	28.72 (28.47 - 28.97)	39.16 (38.52 - 39.79)
**2011**	28.13 (27.89 - 28.38)	38.67 (38.04 - 39.29)
**2012**	28.22 (27.97 - 28.46)	38.17 (37.55 - 38.78)
**2013**	29.08 (28.83 - 29.32)	37.03 (36.43 - 37.63)
**2014**	28.54 (28.3 - 28.78)	36.45 (35.85 - 37.04)
**2015**	30.64 (30.39 - 30.89)	38.47 (37.86 - 39.07)
**2016**	34.74 (34.49 - 35)	42.72 (42.08 - 43.36)

**2017**	37.64 (37.38 - 37.91)	47.18 (46.51 - 47.84)
**2018**	38.85 (38.58 - 39.11)	49.37 (48.7 - 50.05)
**2019**	39.98 (39.71 - 40.25)	52.28 (51.59 - 52.97)
**2020**	55.32 (55.01 - 55.63)	69.88 (69.08 - 70.68)

Supplemental Table 8: Age-Adjusted Mortality Rates per 100,000 for Cardiovascular Deaths (CVD) Related Mortality among adults with T2DM in the United States, 1999 to 2023

Year	Age-Adjusted Rate (95% CI)
1999	9.08 (8.94 - 9.22)
2000	9.49 (9.35 - 9.64)
2001	9.75 (9.61 - 9.9)
2002	10 (9.86 - 10.15)
2003	10.12 (9.97 - 10.26)
2004	10.14 (10 - 10.29)
2005	10.43 (10.29 - 10.58)
2006	10.05 (9.91 - 10.19)
2007	9.93 (9.79 - 10.07)
2008	9.92 (9.78 - 10.06)
2009	9.8 (9.67 - 9.94)
2010	9.58 (9.45 - 9.71)
2011	9.16 (9.04 - 9.29)
2012	9.18 (9.05 - 9.31)
2013	9.28 (9.16 - 9.41)
2014	9.13 (9.01 - 9.26)
2015	9.68 (9.56 - 9.81)
2016	10.94 (10.8 - 11.07)

**2017**	11.77 (11.63 - 11.9)
**2018**	12.2 (12.06 - 12.33)
**2019**	12.47 (12.33 - 12.6)
**2020**	14.58 (14.43 - 14.73)
**2021**	15.96 (15.81 - 16.12)
**2022**	16.08 (15.93 - 16.23)
**2023**	15.78 (15.63 - 15.93)
